# Breastfeeding for reducing the risk of pneumonia morbidity and mortality in children under two: a systematic literature review and meta-analysis

**DOI:** 10.1186/1471-2458-13-S3-S18

**Published:** 2013-09-17

**Authors:** Laura M Lamberti, Irena Zakarija-Grković, Christa L Fischer Walker, Evropi Theodoratou, Harish Nair, Harry Campbell, Robert E Black

**Affiliations:** 1 Department of International Health, Johns Hopkins Bloomberg School of Public Health, Baltimore, MD, USA; 2 Department of Family Medicine, University of Split School of Medicine, Split, Croatia; 3 Centre of Population Health Sciences, University of Edinburgh Medical School, Edinburgh, UK; 4 Public Health Foundation of India, New Delhi, India

## Abstract

**Background:**

Suboptimal breastfeeding practices among infants and young children <24 months of age are associated with elevated risk of pneumonia morbidity and mortality. We conducted a systematic review and meta-analysis to quantify the protective effects of breastfeeding exposure against pneumonia incidence, prevalence, hospitalizations and mortality.

**Methods:**

We conducted a systematic literature review of studies assessing the risk of selected pneumonia morbidity and mortality outcomes by varying levels of breastfeeding exposure among infants and young children <24 months of age. We used random effects meta-analyses to generate pooled effect estimates by outcome, age and exposure level.

**Results:**

Suboptimal breastfeeding elevated the risk of pneumonia morbidity and mortality outcomes across age groups. In particular, pneumonia mortality was higher among not breastfed compared to exclusively breastfed infants 0-5 months of age (RR: 14.97; 95% CI: 0.67-332.74) and among not breastfed compared to breastfed infants and young children 6-23 months of age (RR: 1.92; 95% CI: 0.79-4.68).

**Conclusions:**

Our results highlight the importance of breastfeeding during the first 23 months of life as a key intervention for reducing pneumonia morbidity and mortality.

## Background

Pneumonia, the leading cause of child mortality, was responsible for approximately 1.4 million deaths among children < 5 years of age in 2010 [1]. Pneumonia is also a major cause of global morbidity with an estimated 156 million episodes and 14.9 million hospitalizations per year [2,3]. The incidence of pneumonia illness and deaths is marked by a substantial wealth gap, with the majority of morbidity and mortality occurring in developing countries and among the poorest children [4].

Studies suggest that optimal breastfeeding practices, including exclusive breastfeeding during the first six months of life and continued breastfeeding until 24 months of age, are critical for reducing the burden of pneumonia among infants and young children [4-6]. The protective effect of human milk against respiratory infection is attributed to its numerous immunobiological components [7-9].

A systematic review published in 2002, which assessed the optimal duration of breastfeeding for reduction of respiratory illness and mortality, provided support for the global recommendation for exclusive breastfeeding during the first 6 months of life [6]. The objective of this systematic review is to assess and consolidate evidence supporting the protective effects of breastfeeding on pneumonia incidence, prevalence, hospitalizations and mortality among children <24 months of age in developing countries. To achieve this aim, we employed carefully developed and standardized methods of comprehensive systematic review and meta-analysis [10,11]. The results of this review will be utilized to generate Lives Saved Tool (LiST) projections of the potential deaths averted by increasing the coverage of exclusive breastfeeding for the first 6 months of life and continued breastfeeding until 24 months of age [11].

## Methods

We conducted a systematic literature review of studies assessing the risk of pneumonia by varying levels of breastfeeding exposure among infants and young children <24 months of age. We searched for combinations of keywords (breastfeeding, suckle, breast milk, human milk, colostrum, wet nurse, pneumonia, respiratory tract infection, lower respiratory infection, acute respiratory infection and bronchiolitis) in the following electronic databases: Ovid MEDLINE (from 1948 to 2011), EMBASE (from 1980 to 2011) and the Cochrane central register for controlled trials. No limits were applied for language. We conducted our initial search between the 19th and 21st of November 2008 and two updated searches on the 19th of August 2010 and the 14th of March 2011.

All titles/abstracts of retrieved studies were read, duplicates and irrelevant studies were excluded, and the remaining studies were considered for inclusion if they met the following criteria: 1) randomized controlled trial (RCT) or observational study (cohort, longitudinal, case-control or cross-sectional ); 2) study group <2 years of age; 3) study conducted in a developing country [12], a CEE/CIS country, or among indigenous populations of developed countries; 4) provided data assessing levels of suboptimal breastfeeding as a risk factor for one of the following outcomes: pneumonia incidence, pneumonia prevalence, pneumonia (and all-cause) hospitalizations, pneumonia-specific mortality, and all-cause mortality.

If eligibility could not be determined based on title/abstract alone, the full text was obtained for initial screen. Letters to the editor, case reports and review papers were excluded. A sample of items was screened and checked against the inclusion criteria by an independent review author, and full texts of all included studies were subsequently reviewed to confirm inclusion criteria. We included studies focused on respiratory infections defined as either pneumonia or lower respiratory tract infection (LRTI) but excluded studies that only assessed bronchiolitis, bronchitis, tuberculosis, asthma or upper respiratory tract infections (URIs), such as colds or otitis media. If respiratory infections were not defined or URIs and LRTIs were combined, we only included studies where such cases were hospitalized, assuming illness due to acute LRTI. We also excluded studies reporting exclusive breastfeeding for children beyond 6 months of age and studies that did not restrict the allocation of outcomes to *concurrent* breastfeeding status. Furthermore, studies including infants born to HIV-positive mothers were excluded because of the altered immune status of such infants. Papers identified for data abstraction were subjected to a more thorough review, and those with unclear methodology or data in a form that could not be extracted for meta-analysis were excluded if attempts to contact the study authors were unsuccessful.

The review authors, who were not blinded to the study authors or results, extracted data for each outcome by breastfeeding exposure levels classified according to current WHO definitions (Table 1) [13,14]. To account for varying definitions of exclusive breastfeeding over time, we assigned breastfeeding exposure to a WHO category based on the description of the feeding practice, not the authors’ category label. Consequently, we allocated some exclusive breastfeeding labels to predominant breastfeeding, as previously documented [15]. We did not, however, differentiate between exclusive and predominant breastfeeding on the basis of receipt of prelacteal feeds during the first 3 days of life, since this review did not aim to assess early initiation of breastfeeding.

**Table 1:** **Breastfeeding exposures **[15]

**Exposure Category **[13]	Permitted to Receive
Exclusive Breastfeeding	• breast milk from mother or wet nurse or expressed breast milk • NO other liquids or solids except vitamin drops or syrups, mineral supplements, prescribed medicines, or oral rehydration solutions

Predominant Breastfeeding	• breast milk from mother or wet nurse or expressed breast milk • water and water-based drinks NO food-based fluid with the exception of fruit juice and sugar water • vitamin drops or syrups, mineral supplements, or prescribed medicines

Partial Breastfeeding	• breast milk from mother or wet nurse or expressed breast milk • any other liquids or non-liquids, including both milk and non-milk products

No Breastfeeding	• formula and/or animal’s milk • NO breast milk

Any Breastfeeding	• breast milk from mother or wet nurse or expressed breast milk • Includes children exclusively, predominantly, fully, and partially breastfed

Relative risk (RR) and 95% confidence intervals (CIs) were extracted from all included studies; if these effect measures were unavailable, they were generated using reported numerators and denominators in STATA 12.0 [16]. We organized data into relevant age strata (i.e. 0-28 days, 0-5 months, 0-11 months, 6-11 months, 12-23 months, 6-23 months) and excluded studies that grouped analyses across select age categories. The reference categories for RRs were determined based on relevance to age: exclusive, predominant and partial breastfeeding were the reference categories for infants aged 0-5 months; predominant, partial and any breastfeeding were the reference categories for infants aged 0-11 months; and any breastfeeding was the reference category for all age groups extending from ≥6 months of age.

If a given study reported separate effect measures for ages falling within one stratum in our analysis, we conducted fixed effects meta-analysis to pool the effect sizes. We used random effects meta-analysis to combine estimates across studies for each outcome and age category. Meta-analyses were carried out in STATA 12.0 using the meta command [16].

In following CHERG guidelines, we assessed the quality of the pooled effect measures for each outcome by evaluating the contributing studies and quantitative measures. The CHERG grading system assigns a qualitative score (i.e.‘high’, ‘moderate’, ‘low’, ‘very low’) to each effect estimate in order to assess the value of its inclusion in LiST [11]. Self-selection, which occurs when breastfed children are weaned due to repeated illness or poor growth, and reverse causality, which results when breastfeeding is terminated at the onset of respiratory illness, may bias the association between breastfeeding and pneumonia-specific morbidity and mortality [17]; thus, we utilized a previously detailed scoring system to evaluate the presence of such biases in the studies contributing to each pooled effect measure [15]. In brief, the scoring system penalizes a study for failure to incorporate the following methods intended to reduce bias: (1) exclusion of events among neonates < 7 days of age; (2) exclusion of non-singleton births, premature births, low birth weight infants, and infants with congenital abnormalities or other serious illnesses; (3) determination of breastfeeding exposure immediately before event onset, rather than that concurrent with outcome; (4) determination of the association between weaning and illness/poor growth and subsequent exclusion of such infants or young children [17]. See additional file 1 for detailed abstraction information about studies.

## Results

**Figure 1:**
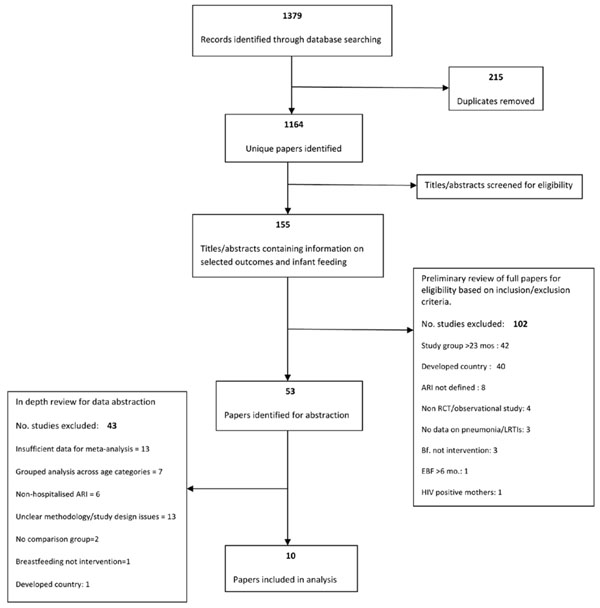
Review process of the effects of suboptimal breastfeeding exposure on pneumonia incidence, prevalence, mortality, hospitalizations, and all-cause mortality and hospitalizations

Our review identified 1164 unique publications. After title and abstract review we fully screened 155 papers (Figure 1). Following in-depth review and data extraction, 10 studies were identified for inclusion in the final analysis [18-27]. Of these, 7 were prospective cohort and 3 were case-control studies. By WHO region, the included studies were conducted in Latin America (n=5), South Asia (n=4), Africa (n=2) and the Western Pacific (n=1), with 1 study located in three different locations.

### Pneumonia incidence

There was a dose-response relationship between level of breastfeeding exposure and the relative risk of incident pneumonia among infants 0-5 months of age. Compared to exclusively breastfed infants 0-5 months of age, the relative risk of incident pneumonia was highest among those not breastfed and lowest among those predominantly breastfed (Table 2). The risk of incident pneumonia was also elevated among not breastfed infants 6-23 months of age (Table 3).

**Table 2:** The effect of suboptimal breastfeeding on selected outcomes during infancy

		0-5 months*			0-11 months*	
**Outcome**	**Reference Category**	**Predominant**	**Partial**	**Not**	**Partial**	**Not**

Pneumonia Incidence	Exclusive	1.79 (1.29-2.48)[27]	2.48 (0.23-27.15)[27]	2.07 (0.19-22.64)[27]	-	-

Pneumonia Prevalence	Exclusive	1.13 (0.25-5.16)[18]	5.45 (1.35-21.97)[18]	5.61 (1.23-25.53)[18]	-	-
	Predominant		4.81 (2.54-9.14)[18]	4.96 (2.07-11.88)[18]		
	Partial			1.03 (0.54-1.95)[18]		

Pneumonia Mortality	Exclusive	1.66 (0.53-5.23)[19,20]	2.50 (1.03-6.04)[19,20]	14.97 (0.67-332.74)[19,20]		
	Predominant		1.37 (0.50-3.78)[20]	13.0 (4.03-42.02)[20]	1.40 (0.91-2.15)[21,22]	2.11 (1.50-2.96)[21,22]
	Partial			9.47 (2.85-31.47)[20]		1.31 (0.9-1.9)[22]

All-Cause Mortality	Exclusive	1.48 (1.14-1.92)[19,20,25]	2.84 (1.63-4.97)[19,20,25]	14.40 (6.13-33.86)[19,20]	-	^-^
	Predominant		1.69 (1.10-2.61)[20]	8.08 (4.45-14.69)[20]		
	Partial			4.77 (2.65-8.61)[20]		

Pneumonia Hospitalization	Exclusive	1.49 (0.80-2.79)[20]	1.54 (0.80-2.98)[20]	4.06 (1.48-11.14)[20]		
	Predominant		1.04 (0.66-1.62)[20]	2.72 (1.12-6.61)[20]	3.44 (1.60-7.37)[26]	8.99 (4.59-17.59)[26]
	Partial			2.63 (1.06-6.53)[20]		2.62 (1.69-4.04)[26]
	Any					4.32 (2.95-6.33)[26]

All-Cause Hospitalization	Exclusive	1.98 (1.25-3.12)[20]	1.88 (1.16-3.04)[20]	6.03 (3.18-11.44)[20]	-	-
	Predominant		0.95 (0.71-1.28)[20]	3.05 (1.82-5.11)[20]		
	Partial			3.21 (1.88-5.49)[20]		

*Effect reported as RR (95% CI)

**Table 3:** The effect of not breastfeeding on selected outcomes in children 6-23 months of age

Outcome*	6-23 months**
Pneumonia Incidence	1.17 (0.37-3.65)[27]

Pneumonia Prevalence	1.93 (1.39-2.69)[18]

Pneumonia Mortality	1.92 (0.79-4.68)[19,23]

All-Cause Mortality	3.69 (1.49-9.17)[19,24]

*No studies reported hospitalizations among infants and young children 6-23 months of age

**Effect reported as RR (95% CI); Any breastfeeding is reference category

### Pneumonia prevalence

The estimated relative risk of prevalent pneumonia was higher among partially (RR: 5.45; 95% CI: 1.35-21.97) and not (RR: 5.61; 95% CI: 1.23-25.53) breastfed infants 0-5 months of age compared to those exclusively breastfed (Table 2). The relative risk of prevalent pneumonia was also elevated among infants 6-23 months of age who were not breastfed compared to those who were (RR: 1.93; 95% CI: 1.39-2.69) (Table 3).

### Pneumonia mortality

The estimated relative risk of pneumonia mortality was higher among partially breastfed (RR: 2.50; 95% CI: 1.03-6.04) compared to exclusively breastfed infants 0-5 months of age, and the same trend was observed among predominantly and not breastfed infants in the same age category (Table 2). In comparing not breastfed to breastfed infants and young children 6-23 months of age, there was also a trend towards higher pneumonia mortality (Table 3).

### All-cause mortality

In comparison to exclusively breastfed infants, the estimated relative risk of all-cause mortality was higher among predominantly (RR: 1.48; 95% CI: 1.14-1.92), partially (RR: 2.84; 95% CI: 1.63-4.97) and not (RR: 14.40; 95% CI: 6.13-33.86) breastfed infants 0-5 months of age (Table 2). The estimated relative risk of all-cause mortality was higher when comparing not breastfed (RR: 3.69; 95% CI: 1.49-9.17) to breastfed infants and young children 6-23 months of age (Table 3). Elevated risk of all-cause mortality was also suggested among predominantly (RR: 1.41; 95% CI: 1.0-1.99), partially (RR: 2.96; 95% CI: 0.75-11.69), and not (RR: 1.75; 95% CI: 0.30-10.26) breastfed neonates compared to those exclusively breastfed.

### Hospitalizations

The estimated relative risk of hospitalization for pneumonia was higher among not breastfed infants 0-5 months of age compared to those exclusively breastfed (RR: 4.06; 95% CI: 1.48-11.14) (Table 2). The estimated relative risk of hospitalization from any cause was higher among predominantly (RR: 1.98; 95% CI: 1.25-3.12), partially (RR: 1.88; 95% CI: 1.16-3.04) and not (RR: 6.03; 95% CI: 3.18-11.44) breastfed infants 0-5 months of age compared to those exclusively breastfed (Table 2).

### Quality assessment and effect size estimates for LiST

Based on the CHERG grading system, all outcomes were moderate in study design and quality (Table 4). There was a consistent trend towards protection conferred by breastfeeding across all outcome-specific estimates. Based on the CHERG standard rules, there is sufficient evidence of the protective effect of exclusive breastfeeding on mortality among infants 0-5 months of age to support its inclusion in *LiST;* the estimates comparing predominant, partial, and no breastfeeding exposure to exclusive breastfeeding on pneumonia mortality will be included in the *LiST* model (Table 5). The final effect size for pneumonia mortality among infants and young children 6-23 months of age was derived from studies with fewer than 50 total events per outcome (Table 5). Thus, according to CHERG standard rule 0, there is inadequate evidence to support the inclusion of these effect sizes in *LiST.*

**Table 4:** Quality assessment of studies measuring the association between suboptimal breastfeeding and selected outcomes

				Directness	
	
No of studies ^(ref)^	Design	Limitations	Consistency	Generalizability to population of interest	Generalizability to intervention of interest
***Pneumonia Incidence: moderate outcome-specific quality***					

1 [27]	Cohort	Reverse causality highly likely (-0.5)	Study shows benefit of EBF among infants 0-5 mos of age; study shows benefit of any BF among infants 6-23 mos of age (+1)	Only Asia (-0.5)	EBF not reported for neonates alone

***Pneumonia Prevalence: moderate outcome-specific quality***					

1 [18]	Cohort	Reverse causality likely (-0.5)	Study shows benefit of EBF among infants 0-5 mos of age; study shows benefit of any BF among infants 6-23 mos of age (+1)	Only Latin America (-0.5)	EBF not reported for neonates alone

***Pneumonia Mortality: moderate outcome-specific quality***					

5 [19-23]	Cohort/Case-control	Reverse causality highly likely or likely for 3 of 5 studies (-0.5)	All studies show benefit of EBF among infants 0-5 mos of age; all studies show benefit of any BF among infants 6-23 mos of age (+1)	Asia, Latin America, Africa, Western Pacific	

***All-Cause Mortality: moderate outcome-specific quality***					

4 [19,20,24,25]	Cohort	Reverse causality highly likely or likely for all 4 studies (-0.5)	All studies show benefit of EBF among infants 0-5 mos of age; all studies show benefit of any BF among children 6-23 mos of age (+1)	Asia, Latin America, Africa	

***Pneumonia Hospitalizations: moderate outcome-specific quality***					

2 [20,26]	Cohort/Case-control	Reverse causality highly likely or likely for both studies (-0.5)	All studies show benefit of EBF among infants 0-5 mos of age; studies show benefit of any BF among children 0-11 mos of age (+1)	Asia, Latin America, Africa	EBF not reported for neonates alone; BF not reported for children >11 mos

***All-Cause Hospitalizations: moderate outcome-specific quality***					

1 [20]	Cohort	Reverse causality highly likely (-0.5)	Study shows benefit of EBF among infants 0-5 mos of age (+1)	Asia, Latin America, Africa	EBF not reported for neonates alone; BF not reported for children >6 mos

**Table 5:** Application of standardized rules for choice of final outcome to estimate effect of breastfeeding on the reduction of pneumonia mortality

	Outcome Measures	Application of Standard Rules
0-5 months*		
**Pneumonia Mortality**	**n=2; 50 events** The risk of pneumonia mortality is 1.66 (0.53-5.23) for predominant BF; 2.50 (1.03-6.04) for partial BF; 14.97 (0.67-332.74) for not BF compared to EBF	**Rule 2: Apply effect estimates**

**6-23 months**		
**Pneumonia Mortality**	**n=2; 28 events** The risk of pneumonia mortality is 1.92 (0.79-4.68) for not BF compared to any BF	**RULE 0: Insufficient Evidence**

*Evaluating events for studies where reference category is EBF

## Discussion

Our findings highlight the protective effects of breastfeeding against pneumonia incidence, prevalence, hospitalizations, mortality and all-cause hospitalizations and mortality. Exclusive breastfeeding conferred incrementally greater protection among infants 0-5 months of age than predominant and partial breastfeeding (Table 2). Furthermore, continued breastfeeding through 23 months of age was protective compared to no breastfeeding (Table 3). These results support the WHO recommendation for exclusive breastfeeding during the first six months of life and continued breastfeeding for 18 months thereafter.

There was a dearth of literature assessing the effect of suboptimal breastfeeding on selected morbidity outcomes during the neonatal period. However, studies reporting the impact of breastfeeding on all-cause mortality were largely consistent.

Our review validates and expands upon the evidence base established by the Lancet nutrition series [5]. We report effect sizes for neonates, where available, and for three additional outcomes—pneumonia prevalence, pneumonia hospitalizations and all-cause hospitalizations. In addition, we used three different reference categories—exclusive, predominant and partial breastfeeding for infants 0-5 months of age. We can therefore comment on the apparent dose-response relationship among infants during this stage of life. Table 2 illustrates an increasing risk of morbidity and mortality across decreasing dose of breastfeeding among infants 0-5 months of age.

The majority of studies included in this review did not utilize methods to reduce reverse causality bias (Table 4). However, the effect sizes were large and consistent across outcomes and age groups and it is therefore improbable that such bias is completely accountable for our findings. This assertion is supported by repeat analyses conducted by four included studies, which report effect sizes of the same direction and comparable magnitude before and after adjusting for reverse causality [20-22,25]. Furthermore, findings were consistent over a wide geographic area.

Our analyses were limited by the inclusion of effect measures calculated with raw data, unadjusted for potential confounders of breastfeeding and illness, such as socioeconomic status. In addition, included studies were observational and thus confounding may be present. Nevertheless, this methodology has been utilized in previous studies and was not a major limitation to our analyses given the consistency across included studies with and without adjustment for confounding. Moreover, poverty has been linked to longer duration of breastfeeding in developing countries, and thus the failure to adjust for certain confounders may have resulted in a conservative estimate of the protection conferred by breastfeeding exposure [28].

This review did not aim to estimate the risk of suboptimal feeding practices among children born to HIV-infected mothers and is therefore limited by the inability to generalize our findings to such populations. Several studies suggest the benefits of exclusive breastfeeding among children born to HIV-infected mothers during the first six months of life [29,30], and the current WHO/UNICEF recommendation supports this practice and continued feeding during the first year in conjunction with ARV drugs during the breastfeeding period [31]. However, further research is necessary in order to confirm the relevance of our reported effect sizes among HIV-infected mothers and infants.

Our findings represent the best available estimates for the protection conferred by optimal breastfeeding against pneumonia and all-cause morbidity and mortality in low- and middle-income countries. The recommendations summarized in Table 5 will therefore be implemented in the *LiST* model.

## Conclusions

This review underscores the essential role of breastfeeding in the prevention of pneumonia-specific and all-cause morbidity and mortality. Our findings highlight the importance of exclusive breastfeeding during the first six months of life and continued breastfeeding for 18 months thereafter for child survival. While we report the best available effect estimates for inclusion in *LiST*, updated systematic literature review and meta-analyses should be repeated periodically, in light of changing patterns of pneumonia and all-cause morbidity and mortality among children 0-23 months of age. This review did not assess the impact of breastfeeding promotion; however, such research is warranted given the low coverage of exclusive and continued breastfeeding in developing countries.

## Competing interests

The authors have no competing interests.

## Authors' contributions

LML contributed to the abstraction of data from included studies, conducted data analyses and led manuscript preparation. IZG led the systematic review and contributed to manuscript preparation. ET and HN assisted the literature review and abstraction. HC oversaw the literature review and abstraction. CLFW and REB provided technical leadership and assisted with the interpretation of the analysis and the final manuscript preparation.

## Supplementary Material

Additional file 1 The ‘Data Abstraction’ tab includes all data abstracted from studies, as well as notes on methodology and limitations. The ‘Reverse Causality’ tab includes the assessment sheet used to systematically score studies on reverse causation bias. Click here for file

## Declarations

The publication costs for this supplement were funded by a grant from the Bill & Melinda Gates Foundation to the US Fund for UNICEF (grant 43386 to "Promote evidence-based decision making in designing maternal, neonatal, and child health interventions in low- and middle-income countries”). The Supplement Editor is the principle investigator and lead in the development of the Lives Saved Tool (LiST), supported by grant 43386. He declares that he has no competing interests.

This article has been published as part of *BMC Public Health* Volume 13 Supplement 3, 2013: The Lives Saved Tool in 2013: new capabilities and applications. The full contents of the supplement are available online at http://www.biomedcentral.com/bmcpublichealth/supplements/13/S3.
